# Terapeutic challenge: vena cava filter retrieval four years after implantation

**DOI:** 10.1590/1677-5449.202301382

**Published:** 2025-03-14

**Authors:** José Júlio Bechir Maués, Karen Falcão Britto, Sara Oliveira Rocha

**Affiliations:** 1 Hospital Porto Dias, Belém, PA, Brasil.

**Keywords:** vena cava thrombosis, vena cava filter, deep vein thrombosis

## Abstract

Vena cava filters are used to treat deep vein thrombosis and pulmonary embolism. Despite the extensive literature on these filters, there is still no reliable evidence that they improve clinical results or mortality in patients with deep vein thrombosis. There are also increasing reports of complications from indiscriminate use, with a complication rate of approximately 19%. Complications include penetration into the vein wall, involvement of adjacent organs, fracture, embolization of filter fragments, and deep vein thrombosis. We describe the successful removal of a vena cava filter 4 years after implantation for inferior vena cava thrombosis. The procedure was performed using common endovascular surgery devices.

## INTRODUCTION

Vena cava filters (VCF) are part of the therapeutic arsenal for deep vein thrombosis (DVT) and pulmonary embolism. The use of VCF in patients with contraindications to anticoagulation is well established in the literature and follows current guidelines.^1-4^ However, the “prophylactic” use of VCF in patients at risk of pulmonary embolism, with or without a history of DVT, remains uncertain and unclear.^4^

Non-randomized studies have shown that VCF contribute to lower mortality and increased pulmonary embolism-free survival in cancer patients with DVT, in addition to reducing the risk of bleeding associated with anticoagulant use, despite increasing the risk of DVT.^4-6^

Other studies have found conflicting results, showing little or no benefit from VCF. In addition, unfavorable outcomes have been demonstrated in patients who undergo VCF placement, including increased mortality and health care costs.^7,8^ Studies evaluating the effectiveness of VCF for DVT treatment and pulmonary embolism prevention are not standardized, since they involve heterogeneous groups of patients.^4^ There are also increasing reports of complications associated with indiscriminate VCF use, with rates reaching around 19%.^1,2^

## PART 1 - CLINICAL SITUATION

A 47-year-old male patient was admitted to the emergency room with severe pain and edema in the lower limbs, associated with incapacitating pain when standing and walking. The patient denied comorbidities, except for previous DVT in the left lower limb (iliofemoral) 4 years ago, attributed to prolonged bed rest involved in herpes zoster treatment.

At that time, the patient underwent pharmaco mechanical thrombectomy, stent implantation in the left common iliac vein, and temporary VCF placement. The VCF was not removed after the procedure. Since then, the patient had been using rivaroxaban irregularly.

On admission, venous Doppler showed acute thrombosis of the femoral, the common femoral, and the iliac veins, and the inferior vena cava. An infrarenal VCF and a thrombosed stent were identified in the left iliac vein. Physical examination revealed significant edema throughout the lower limbs that was associated with hyperemia. The pulse in each lower limb was palpable.

The clinical case was submitted to the Hospital Porto Dias Research Ethics Committee (approval no. 6834222). Written informed consent was obtained according to Declaration of Helsinki recommendations.

## PART 2 - TREATMENT

**Figure 1:**
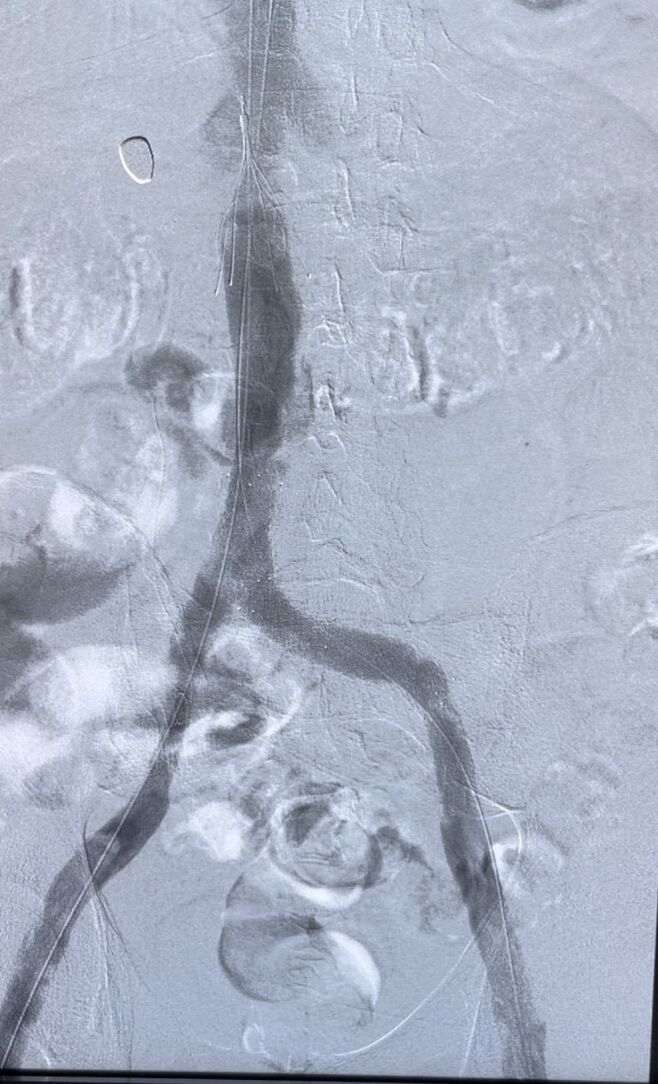
Phlebography with stenoses.

Pharmaco mechanical thrombectomy was performed on the femoral, common femoral, iliac and inferior vena cava veins up to the level of the VCF. Venous recanalization was observed, but with significant stenosis at the level of the VCF, at the proximal edge of the previously implanted stent in the left common iliac vein (which affected more than 50% of the inferior vena cava). Stenosis was also found in the right common iliac vein (Figure 1). Due to the prolonged procedure time and the use of contrast and hemoglobinuria, it was decided to remove the VCF and treat the stenoses in a second stage.

Renal function worsened, with serum creatinine levels of 4.6 mg/dL. During this period, there was complete improvement of lower limb edema, allowing pain-free walking. After creatinine levels normalized, we scheduled the second stage – VCF removal and angioplasty.

The entire procedure was performed under general anesthesia, including puncture of the right and left femoral veins. A 14 F Sentrant sheath (Medtronic, Dublin, Ireland) was used in the right femoral vein, and a short 7 F sheath was used in the left femoral vein. Through a right jugular puncture, a 24 F Sentrant sheath (Medtronic) was placed and, coaxially, a longer 14 F DrySeal sheath (WL Gore and Associates, Newark, DE, USA) was placed.

**Figure 2:**
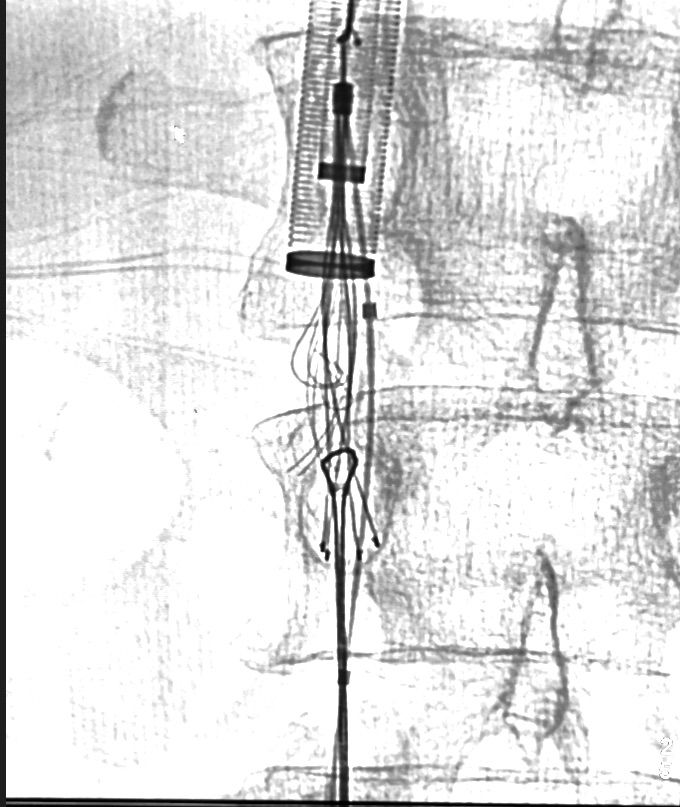
Upper and lower filter capture.

The VCF removal technique included capturing the VCF legs with a snare via femoral access and the upper loop of the VCF via jugular access (Figure 2). The goal was to close and progressively separate the VCF from vena cava wall.

**Figure 3:**
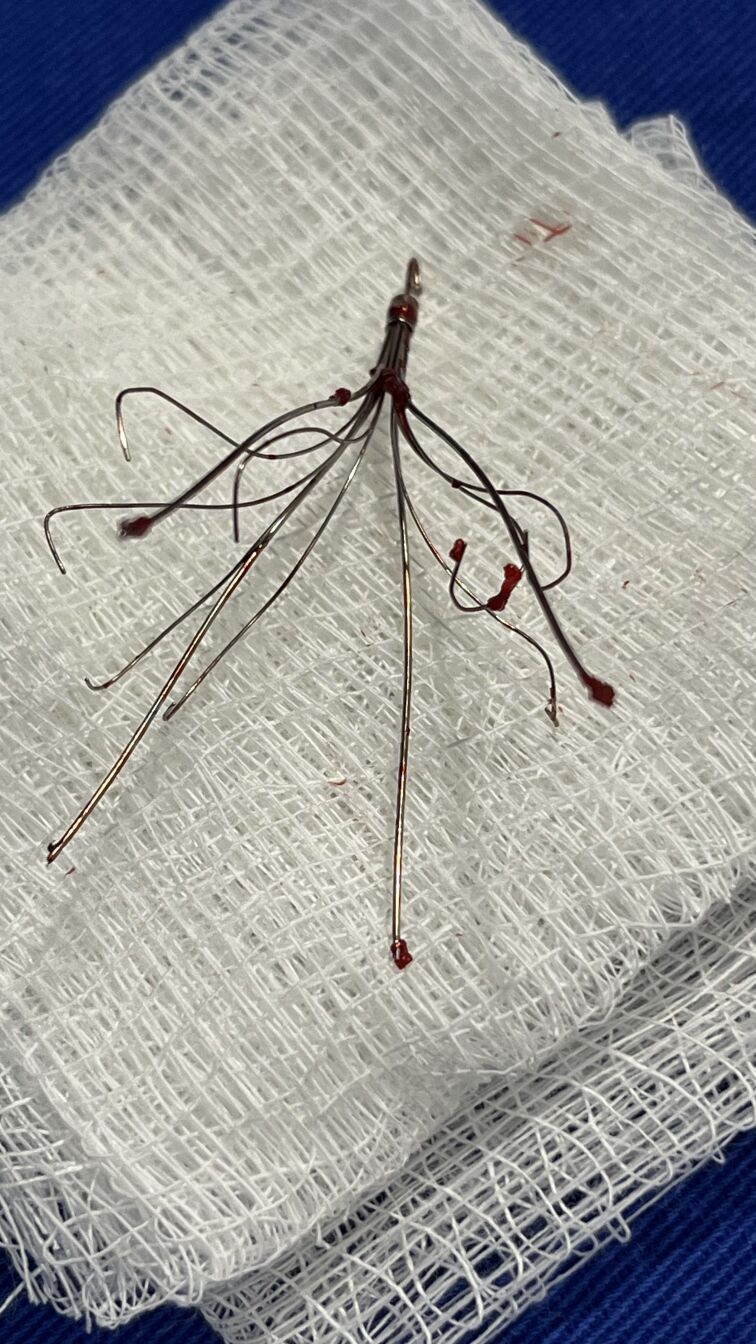
Vena cava filter after removal.

**Figure 4:**
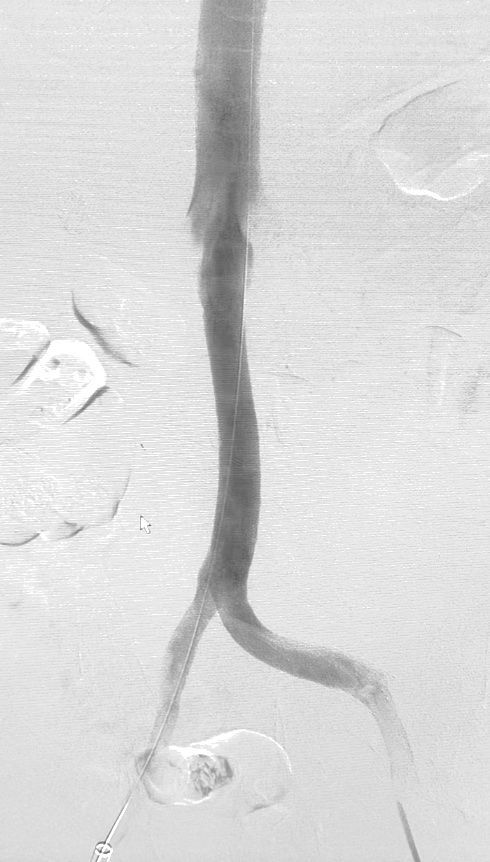
Final phlebography.

A traction-countertraction maneuver was performed with the snare catheters through the femoral and jugular accesses, simultaneously, associated with progression of the DrySeal sheath, keeping the VCF steady to avoid injuring the venous wall. The DrySeal sheath was also rotated to help release the device. With this maneuver the sheath was fully advanced over the VCF. The filter was removed intact (Figure 3). Control angiography showed no contrast medium extravasation (Figure 4). Furthermore, no stenoses previously visualized in the VCF topography or in the stent were identified. Therefore, balloon angioplasty was performed only on the right external iliac vein stenosis, with good angiographic results; no stent was required.

**Figure 5:**
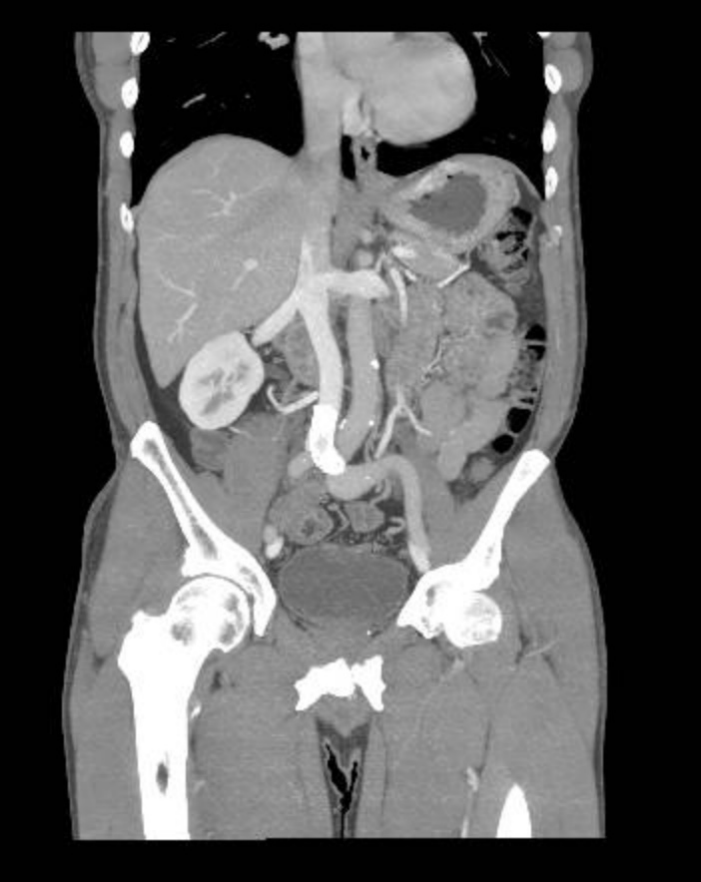
Control venous angiotomography after 3 months.

The patient was discharged on the second day after the procedure, and aspirin 100 mg and rivaroxaban 20 mg per day were prescribed. When he returned after 1 week, he was completely asymptomatic, with complete improvement of the lower limb edema. In the return visit after 3 months, he remained asymptomatic, with venous angiotomography showing patency of the femoral, iliac veins and vena cava axis (Figure 5).

## DISCUSSION

VCF are used to prevent pulmonary embolism in cases where anticoagulation is impossible during lower limb venous thrombosis or as a preventive measure against pulmonary embolism in surgical procedures.

Prolonged VCF dwell time can lead to complications, such as fracture, filter fragment embolization, filter leg perforation of vena cava wall and a new thrombosis or vena cava thrombosis. Thrombosis can lead to post-thrombotic syndrome that could impair the patient's quality of life and can reach up to 90% incidence if untreated. Venous ulcers can occur in up to 15% of the cases, while venous claudication has been observed in 45% of the cases.^9,10^

The incidence of inferior vena cava thrombosis associated with the presence of VCF ranges from 1% to 31%.^9-11^ Ming et al.^11^ found a 31.1% incidence of inferior vena cava thrombosis, attributing this to wide range of definitions of thrombosis at the VCF level and different filter models. Furthermore, factors such as follow-up duration, the studied population, and associated use of anticoagulants and/or antiplatelet agents also affect the incidence of VCF-related inferior vena cava thrombosis.^11^

The filter tilt exceeding 15º and extended dwelling time, particularly in cases involving temporary filters, are also correlated with an increased risk of inferior vena cava thrombosis at the filter site.^9,11^ Delayed removal of the VCF is also subject to intra- and postoperative complications, such as venous rupture, filter fracture and fragment migration to the heart and lung, and venous dissection. Most complications are due to filter adhesion to the vessel wall and the need for techniques requiring multiple puncture sites.^12,13^

## RISKS AND BENEFITS

The risks arising from this case report include breach of confidentiality and the inadvertent disclosure of the patient's personal data, which has been addressed by removing any data that could identify the patient from the report. However, in addition to benefiting the academic community, scientific dissemination of this case could benefit other patients in similar situations by providing data on aspects not previously addressed in the literature.

## CONCLUSIONS

We conclude that the prolonged use of temporary VCF in a patient without contraindication to full anticoagulation triggered extensive bilateral thrombosis of the aorto-iliac-femoral arterial axis, with disabling clinical repercussions. Therefore, it was necessary to remove the VCF for an adequate therapeutic response and prevent new thrombus formation.
