# Supplementary material for: Terapeutic challenge: vena cava filter retrieval four years after implantation
Source: J Vasc Bras. 2025 Mar 14;24:e20230138. doi: 10.1590/1677-5449.202301382 (PMC11922326; doi:10.1590/1677-5449.202301382)
Supplement: Translation

# Translation: Desafio terapêutico: retirada de filtro de veia cava após 4 anos de implante

**DOI:** 10.1590/1677-5449.202301381

**Authors:** José Júlio Bechir Maués, Karen Falcão Britto, Sara Oliveira Rocha

**Affiliations:** 1 Hospital Porto Dias, Belém, PA, Brasil.

**Keywords:** trombose de veia cava, filtro de veia cava, trombose venosa profunda

## Abstract

Os filtros de veia cava fazem parte do arsenal terapêutico para o tratamento da trombose venosa profunda (TVP) e da embolia pulmonar. Mesmo com vasta literatura a respeito do uso desses dispositivos, ainda não existe evidência confiável de que melhorem os resultados clínicos e a mortalidade em pacientes com TVP. Além disso, são crescentes os relatos de complicações associadas ao uso indiscriminado dos filtros de veia cava, podendo chegar a cerca de 19%, incluindo penetração na parede da veia, acometimento de órgão vizinho, fratura, embolização de fragmento do filtro e TVP. Descrevemos a retirada bem-sucedida de um filtro de veia cava após 4 anos de implante, associado a trombose de veia cava inferior. O procedimento foi realizado com materiais do dia a dia da cirurgia endovascular.

## INTRODUÇÃO

Os filtros de veia cava (FVC) fazem parte do arsenal terapêutico para o tratamento da trombose venosa profunda (TVP) e da embolia pulmonar. O uso do FVC em pacientes com contraindicação à anticoagulação está bem estabelecido na literatura e segue as diretrizes atuais^1-4^ . Porém, o uso “profilático” do FVC em pacientes com risco de embolia pulmonar, com ou sem histórico de TVP, segue incerto e obscuro^4^ .

Estudos não randomizados evidenciaram que o uso do FVC contribuiu para a redução da mortalidade e o aumento da sobrevida livre de embolia pulmonar em pacientes oncológicos com TVP, além de reduzir o risco de sangramento associado ao uso de anticoagulantes, apesar de elevar o risco de TVP^4-6^ .

Outros estudos evidenciaram resultados conflitantes, mostrando pouco ou nenhum benefício com o uso do FVC. Além disso, foram demonstrados desfechos desfavoráveis em pacientes submetidos à colocação do FVC, incluindo aumento da mortalidade e elevação dos custos em saúde^7,8^ .

Assim, não há uma padronização nos estudos que avaliam a eficácia do FVC no tratamento do TVP e prevenção de embolia pulmonar, visto que abrangem grupos heterogêneos de pacientes^4^ .

Além disso, são crescentes os relatos de complicações associadas ao uso indiscriminado de FVC, com taxas que podem chegar a cerca de 19%^1,2^ .

## PARTE 1 - SITUAÇÃO CLÍNICA

Um paciente do sexo masculino, de 47 anos, foi admitido na emergência com dor intensa e edema nos membros inferiores, associados a dor incapacitante em ortostase e deambulação.

O paciente negou comorbidades, exceto TVP prévia no membro inferior esquerdo, ilíaco-femoral, há 4 anos, atribuída a repouso prolongado para tratamento de herpes-zóster.

Naquela ocasião, o paciente foi submetido a trombectomia farmacomecânica, implante de *stent* na veia ilíaca comum esquerda e colocação de FVC temporário. O FVC não foi retirado após o procedimento. Desde então, o paciente vinha fazendo uso irregular de Xarelto.

No Doppler venoso na admissão, havia trombose aguda das veias femorais, femorais comuns, ilíacas e da veia cava inferior. Um FVC infrarrenal e um *stent* trombosado na veia ilíaca esquerda foram identificados.

No exame físico, observou-se edema importante de membros inferiores até a raiz das coxas, associado à hiperemia. Os pulsos dos membros inferiores estavam palpáveis.

O caso clínico foi submetido ao Comitê de Ética em Pesquisa do Hospital Porto Dias (parecer de aprovação n.º 6834222). Foi aplicado o Termo de Consentimento Livre e Esclarecido, conforme preconizado pela Declaração de Helsinque.

## PARTE 2 - O QUE FOI FEITO

Foi realizada trombectomia farmacomecânica de veias femorais, femorais comuns, ilíacas e da veia cava inferior até o nível do FVC. Houve recanalização venosa, porém com estenoses importantes no nível do FVC, na borda proximal do *stent* previamente implantando na veia ilíaca comum esquerda (que invadia em mais de 50% da veia cava inferior). Também foi encontrada uma estenose na veia ilíaca comum direita (Figura 1).

**Figura 1:** Flebografia com as estenoses.

Devido ao tempo prolongado de procedimento, ao uso de contraste e à hemoglobinúria, optou-se por retirar o FVC e tratar as estenoses em segundo tempo.

Houve piora da função renal, com níveis séricos de creatinina de 4,6 mg/dL. Durante esse período, observou-se melhora completa do edema de membros inferiores, permitindo deambulação sem dor. Após a normalização dos níveis de creatinina, agendamos a segunda etapa, para retirada do FVC e realização de angioplastia.

Todo o procedimento foi feito sob anestesia geral, com punção de veias femorais direita e esquerda. Na direita, foi utilizada uma bainha Sentrant (Medtronic) 14 F e, na esquerda, foi utilizado introdutor curto 7 F. Também foi realizada uma punção jugular direita com colocação de uma bainha Sentrant (Medtronic) 24 F e, de forma coaxial, uma bainha DrySeal (GORE) 14 F, mais longa.

A técnica para retirada do FVC incluiu a captura das pernas do FVC com laço por acesso femoral e da alça superior do FVC pelo acesso jugular (Figura 2). O objetivo era o fechamento e o descolamento progressivo do FVC.

**Figura 2:** Captura do filtro superior e inferior.

Foi realizada uma manobra de tração e contratração com os cateteres laço pelos acessos femoral e jugular, simultaneamente, associada à progressão da bainha DrySeal (GORE), mantendo sempre o FVC livre de movimentação a fim de evitar lesão da parede venosa.

Foi realizada também a rotação da bainha DrySeal (GORE) para auxiliar na liberação do dispositivo. Com isso, foi possível avançar completamente a bainha sobre o FVC e retirá-lo intacto (Figura 3). A angiografia de controle não evidenciou extravasamento de contraste (Figura 4). Além disso, não foram identificadas as estenoses previamente visualizadas na topografia do FVC ou no *stent*. Assim, foi realizada angioplastia com balão apenas na estenose da veia ilíaca externa direita com bom resultado angiográfico, sem necessidade de *stent*.

**Figura 3:** Filtro de veia cava retirado.

**Figura 4:** Flebografia final.

O paciente recebeu alta no segundo dia após o procedimento, com prescrição de aspirina 100 mg e Xarelto 20 mg por dia. No retorno após 1 semana, estava completamente assintomático, com melhora total do edema de membros inferiores. Permaneceu assintomático no retorno de 3 meses, com angiotomografia venosa mostrando perviedade do eixo cavo-ilíaco (Figura 5).

**Figura 5:** Angiotomografia venosa de controle após 3 meses.

## DISCUSSÃO

Os FVC são utilizados para a prevenção de embolia pulmonar em casos de impossibilidade de anticoagulação durante a vigência de trombose venosa em membros inferiores ou como medida preventiva de embolia pulmonar em procedimentos cirúrgicos.

A permanência prolongada do FVC pode ocasionar complicações, como fratura com embolia do fragmento, penetração na parede da veia cava inferior, além de nova trombose ou trombose de veia cava. Nos casos de trombose, a síndrome pós-trombótica pode ser um fator deletério para a qualidade de vida do paciente, com incidência estimada em até 90% dos casos de trombose não tratada. Úlceras venosas podem ocorrer em até 15% dos casos, enquanto claudicação venosa é observada em 45% dos casos^9,10^ .

A incidência de trombose de veia cava inferior associada à presença de FVC varia de 1 a 31%^9-11^ . Ming et al.^11^ encontraram uma incidência de trombose de veia cava inferior de 31,1% e atribuíram o achado à variabilidade da definição do que seria trombose ao nível do FVC e do tipo de filtro. Além disso, fatores como a duração do acompanhamento, a população e a associação ou não com o uso de anticoagulantes e/ou antiagregantes plaquetários também influenciam a variabilidade da incidência de trombose de veia cava inferior associada ao filtro^11^ .

A inclinação do filtro > 15º e a permanência prolongada, nos casos de filtros removíveis, estão também associadas a uma maior chance de trombose de veia cava no nível do filtro^9,11^ .

A retirada tardia do FVC também está sujeita a complicações intra e pós-operatórias, como ruptura venosa, fratura do filtro com dispersão dos fragmentos para câmara cardíaca e pulmão e dissecção venosa. A maioria das complicações ocorre devido à adesão do filtro à parede do vaso e à necessidade do uso de técnicas que demandam mais de um sítio de punção^12,13^ .

## RISCOS E BENEFÍCIOS

Os riscos decorrentes deste relato de caso incluem a quebra da confidencialidade e a divulgação inadvertida de dados pessoais do paciente, podendo gerar desconforto. Para assegurar que não ocorrerão tais riscos, todos os dados que poderiam identificar o paciente foram retirados da descrição do relato.

Os benefícios da divulgação científica deste caso incluem a contribuição para o paciente ou grupo de indivíduos em situação semelhante, por meio do conhecimento sobre algum aspecto anteriormente não abordado na literatura, além de beneficiar a comunidade acadêmica.

## CONCLUSÃO

Concluímos que o uso prolongado do FVC temporário em um paciente sem contraindicação à anticoagulação plena desencadeou trombose extensa bilateral do eixo cavo-ilíaco-femoral, com repercussões clínicas incapacitantes. Dessa forma, a retirada do FVC foi necessária para proporcionar uma resposta terapêutica adequada e evitar a formação de novos trombos.
